# A Wavelet Neural Network for SAR Image Segmentation

**DOI:** 10.3390/s90907509

**Published:** 2009-09-22

**Authors:** Xian-Bin Wen, Hua Zhang, Fa-Yu Wang

**Affiliations:** 1 Key Laboratory of Computer Vision and System of Ministry of Education, Tianjin University of Technology, Tianjin 300191, China; 2 Tianjin Key Laboratory of Intelligence Computing and Novel Software Technology, Tianjin, 300191, China

**Keywords:** synthetic aperture radar, image segmentation, Wavelet Neural Network

## Abstract

This paper proposes a wavelet neural network (WNN) for SAR image segmentation by combining the wavelet transform and an artificial neural network. The WNN combines the multiscale analysis ability of the wavelet transform and the classification capability of the artificial neural network by setting the wavelet function as the transfer function of the neural network. Several SAR images are segmented by the network whose transfer functions are the Morlet and Mexihat functions, respectively. The experimental results show the proposed method is very effective and accurate.

## 1. Introduction

The synthetic aperture radar (SAR) system is a powerful tool for observing the Earth under all weather conditions. In recent years, SAR imaging has been rapidly gaining prominence in applications such as remote sensing, surface surveillance and automatic target recognition. Segmentation of SAR images is a critical preliminary operation in various SAR images processing applications, such as target detection, recognition, and image compression.

SAR images characteristically have a particular kind of noise, called speckle, which occurs by random interferences, either constructive or destructive, between electromagnetic waves from different reflections in the imaged area. This makes SAR segmentation a difficult task, though several different segmentation methods designed specifically for SAR images have been proposed. Three common methods are optical image segmentation after speckle filter, the multiscale method [1–3], and the neural networks method [4,5].

Artificial neural networks (ANNs) are a class of computational architectures that are composed of interconnected, simple processing nodes with weighted interconnections. Neural networks have proven to be a popular tool for knowledge extraction, pattern matching, and classification due to their capability of learning from examples with both linear and nonlinear relationships between the input and output signals. However, ANNs have limited ability to characterize local features, such as discontinuities in curvature, jumps in value or other edges, so these algorithma are not well suited for speckled SAR images. The wavelet transform, on the other hand, is efficient in representing and detecting local features in images due to the spatial and frequency localization properties of wavelet bases [6]. With the detection of local features, an object can be easily recognized. Many new algorithms based on wavelet transform have been developed to solve SAR image segmentation problems [7,8]. However, the feature-matching of these algorithms have some shortcomings. In order to ensure the reliability of the matching results, they all require an enormous number of scales to construct the time-frequency features at various scales during the classification process. Each scale corresponds to convolving the signal with a wavelet function; hence a large number of convolutions are needed for these algorithms, which make them computationally inefficient.

In this paper, a wavelet neural network (WNN) method is proposed for SAR image segmentation, which takes full advantages of the partial-resolution characteristic of the wavelet transform and the nonlinear mapping behavior of artificial neural networks.

This paper is organized as follows. In the next section, we will describe quadtree interpretation of SAR imagery and its mixture multiscale autoregressive (MMAR) modeling. In Section 3, we will propose a hybrid method based on the GA algorithm and EM algorithm for the MMAR model. In Section 4, we will present the experimental results. In Section 5, we will present a short conclusion concerning our algorithm.

## 2. Wavelet Transform

In signal analysis applications, it is necessary to extract signal features with Fourier transformation, but it is only a time domain transform, which has no time-frequency localization features. The theory of wavelet transformation was first proposed in the field of multi-resolution analysis; among others, it has been applied to image and signal processing. A continuous wavelet transform (CWT) can decompose a signal into a set of finite basis functions, which can uncover transient characteristics in the signal. Wavelet analysis is the breaking up of a signal into dilations and translation versions of the original wavelet, referred to as the mother wavelet. The wavelet must be oscillatory, have amplitudes that quickly decay to zero, and have at least one vanishing moment. Wavelet coefficients *W_x_*(*a*, *τ*) are produced through the convolution of a mother wavelet function *ψ*(*t*) with the analyzed signal *f*(*t*), it is:

(1) $$W_{x}\;(a,\tau )=\frac{1}{\sqrt{\left|a\right|}}\;\int \;f(t)\psi \left(\frac{t-\tau }{a}\right)\mathrm{dt}$$

where *a* and *τ* denote the scale and local center of the analyzing wavelet. By adjusting the scale, *a*, a series of different frequency components in the signal can be obtained.

Several wavelet mother functions have been proposed in the wavelet theory. Each mother function has its suitable application. In this work, the wavelet employed is the Morlet Wavelet, due to its directional selectiveness capability of detecting oriented features, fine tuning to specific frequencies and its good localization in time and frequency [9]. This is a sinusoidal signal modulated by a Gaussian wave. It is characterized for its narrow frequency response, which offers a higher spectral resolution than the Mexican Hat wavelet. This wavelet is particularly useful for filtering out the background noise of the images. In this paper, the Morlet wavelet is applied as:

(2) $$\psi_{a,\tau }\;(t)=e^{-t_{i}^{2}/2}\;\text{cos}\left(5t_{i}\right)$$

where $t_{i}=\frac{t-\tau }{a}$.

## 3. Wavelet Neural Network

### 3.1. Structure of Wavelet Neural Network

The reason for the application of WNN in case of such a problem as classification is that the feature extraction and representation properties of the wavelet transform are merged into the structure of the ANN to further extend the ability to approximate complicated patterns.

The WNN can be considered an expanded perceptron in which the neurons of the first layer are replaced by wavelet nodes [10,11]. The wavelet nodes allow the detection of the transient, as well as the extraction and selection of a small number of meaningful features; the obtained features are then regarded as inputs to the subsequent neurons used as a classifier.

**Figure 1.**
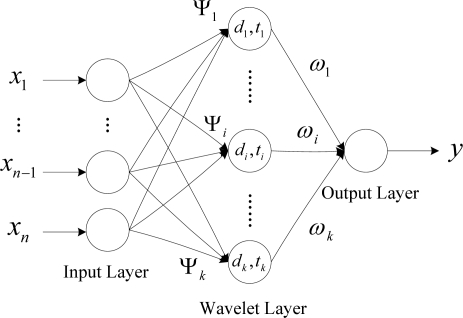
Wavelet Neural Network Structure.

The WNN employed in this paper is designed as a three-layer structure with an input layer, a wavelet layer, and an output layer. The topological structure of the WNN is illustrated in Figure 1. In this WNN model, the hidden neurons have wavelet activation functions of different resolutions and *ω_i_* is the weight connecting the hidden layer and output layer. For an input vector *x* = [*x*_1_, *x*_2_, …., *x*_n_], the output of the *i* th wavelet layer neuron is described as follows:

(3) $$\psi_{k}(x)=\sum_{i=1}^{n}\;\text{exp}\left(-{\left(\frac{x_{i}-d_{k}}{t_{k}}\right)}^{2}/2\right)\text{cos}\left(5\cdot \frac{x_{i}-d_{k}}{t_{k}}\right)$$

where *x_i_* is the *i* th input vector and *k* is the number of wavelet node. *d_k_* and *t_k_* are translation parameter and the dilation parameter, respectively.

The output of the third layer is the weighted sum of *ψ_k_*(*x*)

(4) $$y(x)=\sum_{m=1}^{k}\;\omega_{m}\;\psi_{m}(x)$$

### 3.2. Training of WNN

Wavelet network training consists of minimizing the usual least-squares cost function:

(5) $$E=\frac{1}{2}\sum_{j=1}^{s}\;{\left(y_{j}-o_{j}\right)}^{2}$$

where *s* is the number of training samples for each class and *o_j_* is the optimal output of the *j* th input vector.

Due to the fact that wavelets are rapidly vanishing functions, a wavelet may be too local if its dilation parameter is too small and it may sit out of the domain of interest if the translation parameter is not chosen appropriately.

Therefore, it is inadvisable to initialize the dilations and translations randomly, as is usually the case for the weights of a standard neural network with sigmoid activation function. We use the following initialization procedure, setting.

The same value to dilation parameter *d_k_* is given randomly at the beginning, and initializing the translation parameter *t_k_* is as follows:

(6) $$t_{k}=(k\times s)/K,\;\;\;\;\;\;k=0,1,2\cdots K-1$$

where *s* is the number of training samples for each class and *K* is the number of nodes in the wavelet layer.

The partial derivative of parameters *d*, *t*, *ω* are as follows:

(7) $$\begin{array}{ll} \frac{\partial E}{\partial d_{m}} & =\sum_{j=1}^{s}\;2\left(y_{j}-o_{j}\right)\cdot \left(\sum_{m=1}^{k}\;\omega_{m}\;\text{exp}\left(-{\left(\frac{x-d_{m}}{t_{m}}\right)}^{2}/2\right)\;\left(\left(\frac{x-d_{m}}{t_{m}^{2}}\right)\;\text{cos}\left(5\cdot \frac{x-d_{m}}{t_{m}}\right)+\frac{5}{t_{m}}\text{sin}\left(5\cdot \frac{x-d_{m}}{t_{m}}\right)\right)\right)\\ & =\sum_{j=1}^{s}\;2\left(y_{j}-o_{j}\right)\cdot \left(\sum_{m=1}^{k}\;\omega_{m}\;\text{exp}\left(-\frac{s_{m}^{2}}{2}\right)\;\frac{\left(s_{m}\;\text{cos}\left(5s_{m}\right)+5\;\text{sin}\left(5s_{m}\right)\right)}{t_{m}}\right) \end{array}$$

(8) $$\begin{array}{ll} \frac{\partial E}{\partial t_{m}} & =\sum_{j=1}^{s}\;2\left(y_{j}-o_{j}\right)\cdot \left(\sum_{m=1}^{k}\;\omega_{m}\;\text{exp}\left(-{\left(\frac{x-d_{m}}{t_{m}}\right)}^{2}/2\right)\;\frac{x-d_{m}}{t_{m}^{2}}\;\left(\frac{x-d_{m}}{t_{m}}\;\text{cos}(5\cdot \frac{x-d_{m}}{t_{m}})+5\text{sin}\left(5\cdot \frac{x-d_{m}}{t_{m}}\right)\right)\right)\\ & =\sum_{j=1}^{s}\;2\left(y_{j}-o_{j}\right)\cdot \left(\sum_{m=1}^{k}\;\omega_{m}\;\text{exp}\left(-\frac{s_{m}^{2}}{2}\right)\;\frac{s_{m}}{t_{m}}\;\left(s_{m}\;\text{cos}\left(5s_{m}\right)+5\text{sin}\left(5s_{m}\right)\right)\right) \end{array}$$

(9) $$\frac{\partial E}{\partial \omega_{m}}=\sum_{j=1}^{s}\;\sum_{m=1}^{k}\;\psi_{m}\;2\left(y_{j}-o_{j}\right)$$

where $s_{m}=\frac{x-d_{m}}{t_{m}}$

We adjust the parameters by the following equation:

(10) $$\Theta^{n}=\Theta^{n-1}-\alpha \Delta \Theta$$

where Θ = (*d*,*t*,*ω*)*^T^* is vector of the parameters *d*, *t* and *ω*, *a* is learning rate between 0.1 and 0.9.

## 4. Experiments

**Figure 2.**
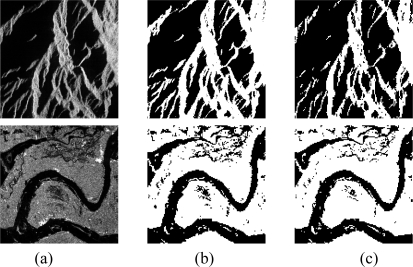
(a) Original SAR image. (b) Segmented image obtained using WNN(Mexihat). (c) Segmented image obtained using WNN(Morlet).

We applied the proposed method to two SAR images sized 256 × 256 pixels [Figure 2(a)] to demonstrate the differences between the Morlet and Mexihat procedures; these images include two regions.

**Figure 3.**
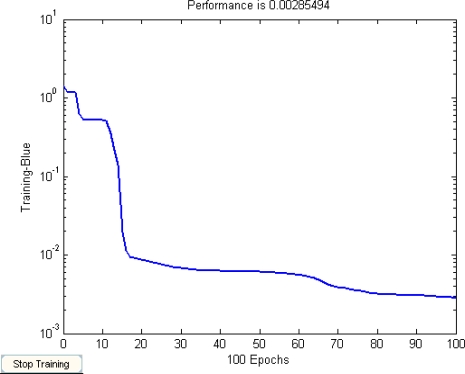
Convergence curve for WNN.

First, about 100 samples were selected as the training data. In our experiment, the initial value of *t_k_* is decided by Equation (6)*d_k_* = 2, and *ω* is a random value between −0.5∼0.5. When the neuron number of the wavelet layer is 25, the segmentation results are best. The compared segmentation results are shown in Figure 2, with (b) showing the Mexihat Wavelet mother function as the transfer function of the second layer and (c) showing the Morlet Wavelet as the transfer function of the second layer. Table 1 is a comparison of the mean square of the above images. It shows that the accuracy ratio of the WNN using Morlet as the transfer function is higher than that of the WNN using Mexihat as the transfer function. Figure 3 is the convergence curve of the WNN training algorithm, which shows the error is almost 10^−2^ by the 25^th^ iteration.

## 5. Conclusions

In this paper, an effective wavelet neural network for SAR image segmentation is proposed. The method not only has the feature of multiscale analysis, but also has a good performance in classification. Experimental results show that using Morlet as the transfer function is better than using Mexihat. WNN is an effective and accurate method for SAR image segmentation.

## Figures and Tables

**Table 1.** Comparison of mean square of the WNN(Mexihat) and WNN(Morlet).

	**WNN(Mexihat)**	**WNN(Motlet)**
Figure 2 (top)	26.256	21.0044
Figure 2 (bottom)	83.69	66.406
